# Exoscope as a Teaching Tool: A Narrative Review of the Literature

**DOI:** 10.3389/fsurg.2022.878293

**Published:** 2022-04-26

**Authors:** Tommaso Calloni, Louis Georges Roumy, Maria Allegra Cinalli, Alessandra Rocca, Andrea Held, Andrea Trezza, Giorgio Giovanni Carrabba, Carlo Giorgio Giussani

**Affiliations:** ^1^Department of Medicine and Surgery, University of Milan Bicocca, Milan, Italy; ^2^Neurosurgery Department, Ospedale San Gerardo, Monza, Italy

**Keywords:** exoscope 3D, exoscope, education, training, resident, students, neurosurgery

## Abstract

Recently, the emergence of the three-dimensional (3D) exoscope has proven to be a viable alternative to the operative microscope (OM) as a novel workhorse of microneurosurgical procedures. Through its current iteration, the 3D exoscope has been demonstrated to be at least equivalent to the operative microscope in terms of surgical outcomes in many settings. With its superior ergonomics and simplicity of use, the 3D exoscope has been shown in multiple studies to be a powerful visualizing tool during surgical procedures. Moreover, the exoscopic systems, through their current iterations and by means of a high-resolution 3D monitor and 3D glasses, have allowed all participants present in the operative room to attain an unprecedented level of intraoperative visualization of anatomical structures and surgical maneuvers which are traditionally available only to the first operator. Although long-term data are still lacking regarding its future as a replacement of the OM, the 3D exoscope has revealed itself as an intense subject of discussion in neurosurgery regarding its implication for surgical education, especially for residents and junior neurosurgeons. This article is a review of the current state of the literature on the role of the exoscope in surgical education, underlining its strength as a learning tool and its potential future implications in terms of surgical education.

## Materials and Methods

We reviewed the English-language literature available on the use of the exoscope as a learning tool, focusing on its involvement in neurosurgical education. Moreover, we used the experience of our center and its use of the three-dimensional (3D) exoscope to guide our review.

### The Benefits of the Exoscope as an Alternative to the OM

The key advantages of the 3D exoscope as an intraoperative visualization tool during neurosurgical interventions are enhanced visualization of the operative field and ergonomics. Its use as an educational tool is another promising feature of the 3D exoscope.

Traditionally, the operative microscope (OM) has been the gold standard for most microneurosurgical procedures. Limitations in magnification and positioning (physical limitations deriving from the lens and the system), poor ergonomics, and the limitation of the surgical view of the first surgeon and the assistant are well-established shortcomings of the OM.

In recent years, a flurry of newer generations of 3D exoscope platforms appeared (ORBEYE, VITOM, MODUS V, KINEVO, and AESCULAP Aeos), each of them with different features and pricing (1, 2).

The exoscope is considered to have excellent vision and ergonomics (3). In contrast to the OM where the surgical assistant is put in an uncomfortable posture, limiting their ability to observe and assist the lead surgeon (4), the exoscope allows for a more relaxed position and greater participation of the surgeon assistant. Besides ergonomic benefits, the ability to generate videos and online views with anatomical detail usually available only to operating surgeons and improved educational value and immersion compared to the OM, to the benefit of non-scrubbed personnel are consistently reported to be the strengths of the exoscope over the OM (5–12).

The exoscope furthermore allows for the execution of variation of traditional approaches and could make less frequent approaches more common, such as the retro-sigmoid approach in the supine position, which the exoscope allows to be carried out in an ergonomically comfortable position (13). This could make learning complex procedures more approachable by reducing physical strain on the surgeon.

The exoscope appears to be well-suited to be integrated with the use of intraoperative ultrasound, a technology that has proved useful in settings, such as adult (14) and pediatric (15) brains and spine (16) surgery. Bulky operative microscope heads often need to be removed from the operative field to apply the ultrasound probe to the tissue, while smaller exoscope cameras, which can sit further away from the operative field, allow for use of ultrasound probe without their removal and, indeed, even with picture-in-picture visualization of the ultrasound images on the exoscope screen (7).

Numerous studies have already pointed out the viability of the exoscope as a visualization device in a multitude of neurosurgical settings, including glioblastoma surgery (17), nerve sheath surgery (18), anterior (5) cervical approaches, anterior lumbar approaches (3), pediatric neurosurgery (although the authors report an instance of switching to the OM over illumination concerns, possibly due to the model of exoscope used) (19), skull base procedures (4), transsphenoidal pituitary procedures (9), as well as many other procedures (20–29).

Concerning vascular surgery, while some authors consider the OM to be preferable namely for aneurysm clipping (28), this is not the case for other authors (30–32).

### Disadvantages in the Use of the 3D Exoscope

The 3D exoscope has only been recently introduced in neurosurgery as a viable alternative tool to the OM. Like its predecessor the OM, it appears to require extensive training and usage to master as enhanced visualization is not synonymous with ease of use.

However, some authors have expressed reserves about the adoption of the exoscope citing resolution, angled view, and costs among the chief concerns (33).

Various studies point out, in particular, an increase in the average duration of neurosurgical intervention with respect to the same intervention conducted with an OM, although the difference in average duration often proves to be not statistically significant (34). Some authors hypothesize that this difference in operative durations can be explained due to a shallower depth of field and constant need for repositioning and refocusing, at least in experimental settings, which might be variable across different brands of exoscopes (8), while others complain the lack of a mouthpiece (30).

It is intuitive a learning curve exists and the exoscope appears to be rated higher by surgeons who are at least somewhat familiar with it: in a study involving both neurosurgeons and otologic surgeons examining the use of the 3D Robotic Digital Exoscope, the exoscope is rated significantly higher by surgeons more familiar with the device (at least 3 procedures) (10). Of note, this is also the case for overall more experienced surgeons (at least 10 years of surgical experience) (10).

Regarding the steepness of the learning curve, diverging opinions exist in the literature, in which a steep learning curve in microvascular anastomosis with the 3D exoscope has been described which is not as fluid as under OM (35). However, other authors describe relatively short learning curves for exoscope adoption by expert vascular surgeons in an experimental setting with 20–30 reported attempts before proficiency with the exoscope was attained by experienced surgeons (36). Moreover, some authors speculate that extensive experience with endoscopic surgery allows for a quicker learning curve with the exoscope in a variety of settings (3, 19).

The actual duration of training to attain surgical proficiency remains to be more definitely investigated, and it remains a question whether the mastery of the exoscope is simpler or harder to attain than that of the microscope for completely inexperienced trainees.

While the exoscope camera is less bulky than a traditional OM and does not require lining up the eyes of the surgeon and assistant to the eyepieces, a further one or 2 high-resolution monitors, up to 55 inches diagonal, need to be set up in the OR. A complete rethinking of the OR set-up to attain an unobstructed view of the monitors (30) is thus often required and another skill to be acquired by the entire team. On the other hand, neurosurgeons appreciate the use of space afforded by the exoscope more than otologic surgeons, an effect the authors speculate to be due to the bulk of neurosurgical OMs compared to nimbler microscopes used in otologic surgery (37).

Visualization angles for the assistant surgeon have drawn some concern, but this can be significantly reduced by the positioning of the exoscope between the surgeon and the surgical assistant and the screen directly in front of the exoscope (38) or through the use of a second screen matching the assistant's point of view with rotated images, especially in spine surgery (2).

Another dubious drawback of the use of exoscopes, which happens to be the other side of the improved ergonomics, is the uncoupling of the surgeon's line of vision from the surgical approach orientation (39), while some authors consider this to be a problem (33), particularly for more experienced surgeons with more consolidated motor schemes (27) this happens to be the very reason for the improved ergonomics and the possibility to achieve very steep angles of vision (27). In the authors' experience, while being unable to rely on core, shoulder, and neck proprioception to help in surgical orientation is a striking difference to the OM, this is overcome quickly.

### The Exoscope as a Potent Tool in Surgical Education

Various studies underline the potency of the exoscope as a surgical learning tool in both simulated and real-life surgical cases.

Since the inception of operating theaters, the importance of watching surgery being performed for trainees has been accepted in the medical community.

It has been reported that one of the main strengths of the exoscope with respect to the standard OM is that it allows all participating staff members present in the operative room to visualize microanatomical details of the surgical field, a level of visualization usually only experienced by the first and second surgeons on operative microscope (5, 10, 25). This tends to be one of the most valued aspects of exoscope use, especially for non-scrubbed-in personnel.

This improved visualization has the potential to revolutionize the way surgical information is conveyed as minute details of the surgical procedure and of the dissection techniques by promoting higher participation of residents, fellows, and as well as scrub nurses (19); this has been apparent since the introduction of the exoscope into the operative setting.

It is furthermore to be noted that the exoscope does not obstruct the view of the lead surgeon's hands, which allows a clear dual perception of the surgical field by the assisting surgeon, and consequently a better orientation on the surgical field. This is particularly true in spinal surgery, which is widely viewed by residents as challenging due to shallow surgical corridors and visualization impediments; in that setting the exoscope solves this issue by allowing high-quality visualization of the operative field and an unobstructed view of the surgeon's hands, instruments, and working angles (25).

However, surgical education is not limited to young neurosurgeons and residents as watching high-resolution surgeries from the first surgeon perspective can prove to be extremely valuable for experienced surgeons as well when it comes to learning about rarer procedures, such as bypass surgery (30).

On the other hand, some authors failed to find a statistically significant difference in self-reported educational usefulness between OM and exoscope. The authors speculate this could be an effect of the highly advanced OM used (Kinevo 900) (40).

### Hands-On Training

Mastery of basic skills was shown to be improved regardless of surgical experience in a study involving 20 neurosurgeons training on a 3D printed model simulating both endoscopic and exoscopic intracerebral hematoma (ICH) evacuation. The training program consisted of the aspiration of a gelatin-like substance simulating a hematoma using the exoscope and the endoscope five times. In this simulated setting, surgery duration and weight of hematoma removed were not significantly different between exoscope and endoscope across the groups of neurosurgeons with different degrees of experience (41).

Indeed, in a laboratory training of sutures, when students and residents were trained on both the exoscope and the microscope, the majority of the trainees (6 out of 8) reported higher ease of use with the exoscope. The authors suggest that seeing the results of one's action on the screen resembles the action of playing video games, underlining that one of the users who preferred the microscope reported not playing video games (12). As more digital natives enter the surgical profession, these seem to be a consideration worthy of further investigation.

Concerning hands-on training, the value of exoscope as a teaching tool for residents was underlined also by cadaver dissection studies (42), providing a safe setting for exploring eye-hand coordination on anatomical structures.

The value of exoscope as a tool for young neurosurgical trainees has been explored in a study investigating carrying out the evacuation of an ICH conducted under the supervision of a more experienced surgeon (43). This neurosurgical procedure was selected being the more accessible of neurosurgical intervention requiring the use of magnification. Due to its ergonomics, the exoscope was noted to afford the possibility to perform a four-handed procedure, with the supervisor managing the exoscope to ensure correct placement of the lens to optimize the surgical field of view. This study concluded the feasibility of conducting ICH evacuation by young neurosurgical using the exoscope; comfortable positioning and that shared field of view are definitely advantages favoring the use of the exoscope as a learning surgical tool. Moreover, it seems to us that another added advantage is the possibility for the teaching surgeon to comfortably step in at any time of the procedure to help the resident without needing to operate from an uncomfortable position or to lose time moving around the microscope should a problem requiring the expert surgeon to step in occur, thus possibly increasing the confidence in allowing inexperienced trainees to start carrying out surgical procedures. All of this could incentivize a greater and earlier resident involvement in microneurosurgical procedures without impact on patient safety.

## Conclusion

Ergonomics and an unobstructed surgical field are the main advantages over the OM. Due to the high-resolution monitors conveying minute anatomical details and unobstructed view of the field and surgeon's hands, high participation levels from residents are expected.

While the exoscope appears to be a promising tool for both neurosurgical practice and neurosurgical education, the overall educational benefits remain to be explored and quantified through future studies, as is the learning curve of the exoscope and the viability of exoscope training independently (i.e., in parallel, or even before) OM training.

Steep adoption costs and the alternative use of the OM might prevent or at least delay widespread adoption of this technology. As of the writing of this article, industry sources for the ORBEYE described fewer than 40 units in use throughout Europe, and the available literature when the first author institution was considered were overwhelmingly from high-income countries.

## Author Contributions

TC and LR: study conception, literature review, and manuscript drafting and revision. MC, AR, and AH: literature review and manuscript drafting. AT: literature review and manuscript drafting and revision. GC: manuscript revision and scientific oversight. CG: study conception, manuscript revision and study oversight. All authors approved the final version of the manuscript.

## Conflict of Interest

The authors declare that the research was conducted in the absence of any commercial or financial relationships that could be construed as a potential conflict of interest.

## Publisher's Note

All claims expressed in this article are solely those of the authors and do not necessarily represent those of their affiliated organizations, or those of the publisher, the editors and the reviewers. Any product that may be evaluated in this article, or claim that may be made by its manufacturer, is not guaranteed or endorsed by the publisher.
